# Evaluation of Female Recipient Infertility and Donor Spermatogonial Purification for Germ Cell Transplantation in *Paralichthys olivaceus*

**DOI:** 10.3390/ani14192887

**Published:** 2024-10-08

**Authors:** Yuqin Ren, Yuehong Tao, Zhaohui Sun, Yufen Wang, Weidong Li, Zhongwei He, Guixing Wang, Yucong Yang, Jilun Hou

**Affiliations:** 1 Hebei Key Laboratory of the Bohai Sea Fish Germplasm Resources Conservation and Utilization, Beidaihe Central Experiment Station, Chinese Academy of Fishery Sciences, Qinhuangdao 066100, China; renyq@bces.ac.cn (Y.R.); m18715539385@163.com (Y.T.); sunzh@bces.ac.cn (Z.S.); wangyf@bces.ac.cn (Y.W.); hezw@bces.ac.cn (Z.H.); wanggx@bces.ac.cn (G.W.); yangyc@bces.ac.cn (Y.Y.); 2 Bohai Sea Fishery Research Center, Chinese Academy of Fishery Science, Qinhuangdao 066100, China; 3 Shanghai Collaborative Innovation for Aquatic Animal Genetics and Breeding Genetics, Shanghai Ocean University, Shanghai 201306, China; 4 Tangshan Haidu Aquatic Food Co., Ltd., Tangshan 063000, China; weidong1970@hotmail.com

**Keywords:** ovarian depletion, spermatogonia stem cell, transplantation, *Paralichthys olivaceus*, busulfan

## Abstract

**Simple Summary:**

Germ cell transplantation (GCT) has been widely used in fish breeding and genetic resource conservation. However, the technique of spermatogonial stem cell transplantation (SSCT) using female adults as recipients has not been established in *Paralichthys olivaceus*. There are two key points of GCT technology, one is the preparation of sterile recipients and the other is obtaining a high proportion of donor germ stem cells. We found that three-year-old fish were the most suitable recipients through the use of a combination of high temperature and busulfan. The proportion of spermatogonial stem cells (SSCs) after isolation and purification was increased by more than 60.00%, and they also successfully colonized and proliferated in the recipient ovaries. The results of this study lay a foundation for the subsequent establishment of SSCT in *P. olivaceus* using female adults as the recipients and provide a reference for using GCT in other fish.

**Abstract:**

Since the advent of germ cell transplantation (GCT), it has been widely used in shortening the fish breeding cycle, sex-controlled breeding and the protection of rare and endangered fish. In this study, the effectiveness of female sterile recipient preparation and donor stem cell isolation and purification were comprehensively evaluated for spermatogonial stem cell transplantation (SSCT) in *Paralichthys olivaceus*. The best way to prepare sterile recipients was found to be giving three-year-old fish four intraovarian injections of busulfan (20 mg/kg body weight) combined with exposure to a high temperature (28 °C) after the spawning season compared with the two other ways, which induced apoptosis of most of the endogenous germ cells, resulting in shrinkage of the spawning plate and enlargement of the ovarian lumen. Further analysis showed that both the gonadosomatic index and germ-cell-specific *vasa* expression were significantly lower than those of the natural-temperature group before treatment (*p* < 0.05). A high percentage (>60.00%) of spermatogonial stem cells (SSCs) were obtained after isolation and purification and were transplanted into the prepared recipients. After three weeks of SSCT, the numbers of PKH26-labeled SSCs were increased in the ovaries of the recipients. These findings provide a basis for the establishment of an ideal SSCT technique using *P. olivaceus* females as the recipients, ultimately contributing to the efficient conservation of male germplasm resources and effective breeding.

## 1. Introduction

Germ cell transplantation (GCT) is a technique in which the recipient acts as a surrogate to produce the progeny of the donor. Since the establishment of a GCT system in rodents [1], it has been used successfully in goats, pigs, monkeys, and other animals [2,3]. The first application in fish was in *Oncorhynchus mykiss* in 2003 [4]. Initially, both intra-species and inter-species GCT were performed using primordial germ cells (PGCs) from donors [5,6]. However, due to the relatively small numbers of PGCs and difficulties in their extraction, the use of GCT in fish was limited until the breakthrough of the development of spermatogonial cell transplantation in *Oncorhynchus mykiss* [7]. Currently, GCT in fish mainly involves the use of spermatogonial stem cells (SSCs) or oogonial stem cells (OSCs) of donors instead of PGCs, which has significantly shortened the fish breeding cycle, enabled sex-controlled breeding and the conservation of rare and endangered fish species [8,9,10,11]. Both SSCs and OSCs have the same sexual plasticity as PGCs, allowing them to develop into either gender depending on the environment of the recipient gonad. This feature allows for various reproductive operations, such as obtaining Y eggs from SSCs transplanted into sterile female recipients for producing YY supermales [12] or the effective preservation of male germplasm resources by transplanting SSCs into recipient gonads of both sexes [13,14]. Similar advantages have been seen for OSCs [15,16].

Germ cells are usually transplanted into the abdominal cavity of hatchling fish [14,17], embryos [5,7], or sterile adult gonads [16] of recipients (closely related species), so that the recipients produce the offspring of the donors. Adult fish, as recipients of GCT, can produce offspring in the second breeding season, allowing for the acceleration of the breeding process [16] and the conservation of germplasm resources [18]. This is especially useful for endangered fish species [19] and has thus attracted much attention from researchers. To use adults as recipients in GCT, apoptosis is induced in the endogenous germ cells, allowing for the development and proliferation of the donor germ cells; this is usually achieved through chemotherapy drug treatment [20,21,22,23], hybridization [24], gene editing [1,25], irradiation [26], or high-temperature treatment [23,27]. Because the preparation of gene-edited fish requires at least two generations and irradiation is not very realistic because fish cannot stay out of water for too long, the combination of a drug and heat treatment is more convenient and rapid for the preparation of sterile recipient fish. This has been applied in *Cyprinus carpio* [27], *Oreochromis niloticus* [22], *Astyanax altiparanae* [23], *Danio rerio* [21], and other fish species.

*Paralichthys olivaceus* is an important aquaculture species in China, Korea and Japan due to its fresh meat, low fat content, and high protein and vitamin content [28,29,30]. It has the advantage that females grow faster than males. However, males are superior to females in some respects. We can perform cold shock-induced androgenesis to fix certain male traits but the induction efficiency is not high [31]. If oocytes derived from spermatogonial stem cell transplantation (SSCT) into females are then used for gynogenesis, it is possible to obtain a new germplasm that grows rapidly and carries superior male traits. The two key factors in the use of SSCT in female fish are the preparation of sterile recipients and the isolation and purification of SSCs. Therefore, the present study evaluated the extent of endogenous germ cell apoptosis of one-, two-, and three-year-old recipient fish injected with busulfan in different ways to select the optimal method for recipient preparation, together with assessing the rate of SSC acquisition of one-year-old donors after enzyme digestion and Percoll gradient centrifugation; finally, we transplanted SSCs into sterile recipient gonads to evaluate the levels of colonization and proliferation.

## 2. Materials and Methods

### 2.1. Experimental Fish

The experimental fish were all obtained from Beidaihe Central Experimental Station, Chinese Academy of Fishery Sciences. Female fish of three ages (one, two, and three years old) were used for preparing recipients. Before each recipient treatment experiment, 50 fish of the same size were transferred into a 25 m^3^ experimental tank and maintained for one week to allow adaptation to the experimental environment. The one-year-old males were temporarily kept in a 3 m^3^ tank for the preparation of donor SSCs. Basic data, such as the body weight and development stage of experimental fish of all ages, were recorded (Table 1).

### 2.2. Heat and Busulfan Treatment for Recipient Fish

The water temperature of recipient fish was gradually increased to 28 °C using an increase of 1 °C per day. In the spawning season (between February and May), the 1-year-old fish were farmed in water at 28 °C for 1.5 months, and then received two injections of busulfan (Macklin, B803921, Shanghai, China), the first was an intraperitoneal injection (40 mg/kg body weight) and the second was administered using an enema tube (length 12 cm, inner diameter 2–3 mm) directly targeted to the ovarian lumen through the urogenital aperture and fallopian tube (18 mg/kg body weight), referred to as intraovarian injection. In the same season, the 2-year-old fish were maintained at 28 °C for two months, and then received two intraovarian injections with a dose of 18 mg/kg body weight of busulfan. After the spawning season (between July and September), the 3-year-old fish were acclimatized to the high temperature for one week, and then received four intraovarian injections with 20 mg/kg body weight of busulfan. The intervals between the injections were two weeks in all three treatment (TM) groups. Additional groups were set up at the same time, namely, the natural-temperature control group (NT group, 18–22 °C), the high-temperature control group (HT group, 28 °C), and the negative control group (NC group; injected with the same volume of DMSO (Macklin, D806645, Shanghai, China) as the TM groups in 28 °C). Two weeks after the last injection, survival rates were measured and three samples were randomly collected from each group. The gonad samples were fixed with 4% paraformaldehyde (Servicebio, G1101, Wuhan, Hubei, China) for histological and immunofluorescence analysis and frozen in liquid nitrogen for Western blotting and quantitative RT-PCR analyses. At the same time, both body and gonad weights were recorded.

### 2.3. Preparation SSCs from Donors

Spermatogonia from a one-year-old male *P. olivaceus* were isolated, purified, and analyzed to obtain a high proportion of SSCs for subsequent transplantation experiments. The testis cells were digested for 3 h (0.25% trypsin, Worthington, LS004454, Lakewood, New Jersey, USA; 0.05% DNase, Roche, 11284932001, Basel, Basel-Stadt, Switzerland), filtered (40 μm cell filter, Sangon, F613461-9001, Shanghai, China), and the Percoll gradient centrifugation was performed (20%, 35%, 50% from top to bottom, 800× *g*, 30 min) for spermatogonia purification according to the methods of oogonia purification in *P. olivaceus* previously described [29]. Residual 0.5 cm^3^ tissues and cell samples were fixed with 4% paraformaldehyde for subsequent analysis. Four biological replicates were used in all cases. Lastly, the purified SSCs were labeled with a 10 μmol working solution of PKH26 (Sigma-Adlrich, MINI26-1KT, Saint Louis, MI, USA) for 5 min, washed twice with PBS (Solarbio, G4202, Beijing, China), and resuspended to 1 × 10^7^ cells/mL for transplantation.

### 2.4. Transplantation and Post-Transplantation Evaluation

After the preparation of the recipients, the temperature was gradually reduced from 28 °C to 20 °C at a rate of 1 °C per day. To test whether the SSCs could colonize and proliferate in the prepared three-year-old recipients, the fish were then placed on the operating platform and injected with 0.5 mL cell suspension (about 5 million cells) through the urogenital aperture and fallopian tube using a 12 cm enema tube (inner diameter 2–3 mm) when the gills did not move after 2–3 min of anesthesia with MS-222 (Macklin, M-E808894, Shanghai, China). The average time for injecting each fish was about 0.5–1 min. After the experiment, the fish were placed in a bath containing 3 ppm penicillin for five days. Gonadal samples were collected and frozen before microscopic evaluation of gonad recovery status and the fate of the PKH26-labeled cells in the recipients 14 and 21 days after SSCT.

### 2.5. Histology and Immunofluorescence Analysis

Cell samples were washed with PBS (Solarbio, G4202, Beijing, China) three times and embedded in agarose for backup. The agarose-embedded and tissue samples were embedded in paraffin, and about 100 continuous sections were made with a thickness of 5 μm each. Some sections were stained with hematoxylin–eosin (HE) and others were subjected to immunofluorescence analysis using anti-Vasa polyclonal antibodies prepared by our team in accordance with our previous immunoassay methods [16] and scanned using a 3D Histech digital section scanning analysis system (Model Pannoramic MIDI, Budapest, Hungary).

### 2.6. Quantitative Real-Time PCR

qRT-PCR primers for *vasa* and *UbcE* were designed using Primer 5.0. The primer sequences are shown in Table 2. RNA extraction was performed using the RNAiso Plus Total RNA Reagent kit (TaKaRa, 9108, Beijing, China). The RNA was reverse-transcribed to cDNA using a PrimeScriptR RT reagent kit with the gDNA Eraser reverse-transcription kit (Takara, RR047A, Beijing, China). The cDNA from the samples was diluted 5–20 times and the expression was analyzed using the SYBR reagent method. Amplification was performed in fluorescent 96-well plates by lightCyCLER480 Real-Time PCR. The reaction system was 20 μL, including 10 µL of 2 × SYBR Premix Ex Taq, 0.4 µL of positive and negative primers, 2 μL of diluted cDNA, and 7.2 µL of ddH_2_O. The fluorescent quantitative PCR reaction was: 95 °C, 30 s; 95 °C, 5 s, 60 °C, 30 s, 40 cycles; 95 °C, 5 s, 60 °C, 60 s. The relative expression of the samples was analyzed by the 2^−ΔΔCT^ method [32].

### 2.7. Western Blotting

Gonadal samples were frozen in liquid nitrogen for 24 h and then stored at −80 °C until required. Proteins were extracted from the samples using RIPA buffer (Servicebio, G2002, Wuhan, Hubei, China) and processed according to standard Western blotting protocols for analyzing Vasa expression during recipient preparation. The primary and secondary antibodies were the anti-Vasa polyclonal antibody, as used for immunofluorescence, and Cy3-conjugated goat anti-rabbit (Servicebio, GB21303, Wuhan, Hubei, China), respectively. β-actin (Servicebio, GB12001, Wuhan, Hubei, China) was used as the loading control.

### 2.8. Statistical Analysis

Single-factor analysis of variance was used for analyzing the differences between different groups followed by least significant difference (LSD) multiple comparisons using SPSS 22.0. The data were presented as mean ± standard error. Statistical significance was indicated when the difference was at *p* < 0.05.

## 3. Results

### 3.1. Survival and Growth after Busulfan Treatment

**Figure 1:**
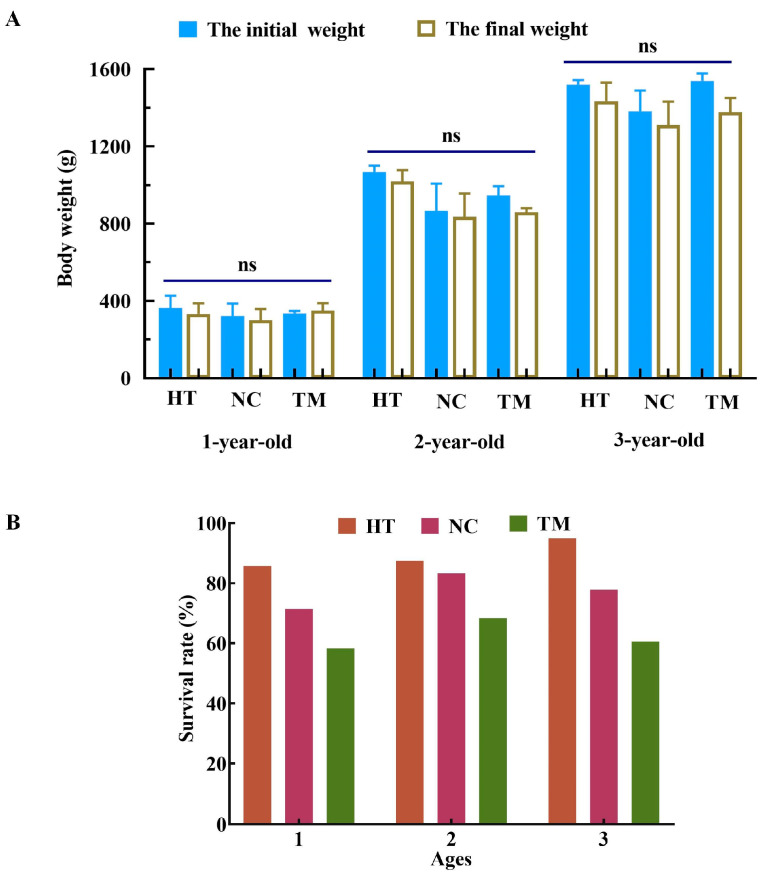
Body weight changes and survival rates in the different groups of one-, two-, and three-year-old fish after recipient treatment. (**A**) Body weight changes in the HT, NC, and TM groups of one-, two- and three-year-old fish before and after treatment. (**B**) The survival rate of one-, two-, and three-year-old fish in the HT, NC, TM groups after treatment. HT group: high-temperature group; NC group: negative control group; TM group: treatment group. ns: no significant difference.

Before busulfan treatment, the body weights of the fish in the one-, two-, and three-year-old groups were 340.00 ± 26.94 g, 960.00 ± 52.55 g, and 1480.00 ± 41.75 g, respectively (Table 1). At the end of the busulfan treatment, almost all groups showed slight decreases in body weight compared with the baseline, but these did not differ significantly from baseline values (*p* > 0.05, Figure 1A). Meanwhile, the survival rates in the three TM groups were 58.3%, 68.4%, and 60.6%, respectively, which were lower than those of the control groups (NT group, HT group, and NC group) of the same age, while there was little variation in survival between the three TM groups (Figure 1B).

### 3.2. Apoptosis of Endogenous Germ Cells in the Recipient after Busulfan Treatment

**Figure 2:**
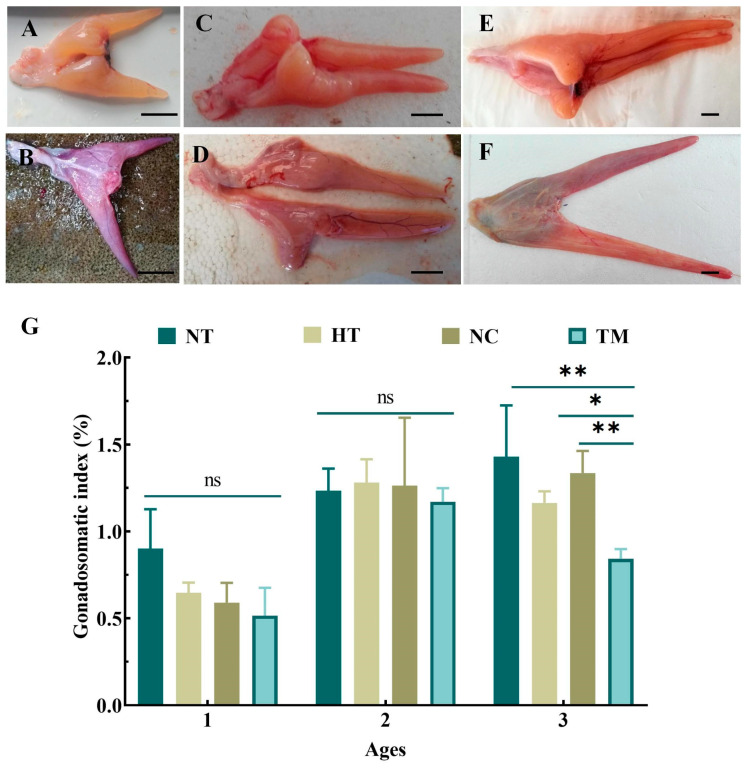
Ovarian atrophy after gonad depletion experiment. Ovarian atrophy in the TM group (**B**) compared with the NT group (**A**) of one-year-old fish. Ovarian atrophy in the TM group (**D**) compared with the NT group (**C**) of two-year-old fish. Ovarian atrophy in the TM group (**F**) compared with the NT group (**E**) of three-year-old fish. The comparison of the gonadosomatic index (%) in the NT, HT, NC, and TM groups of one-, two-, and three-year-old fish (**G**). NT group: natural-temperature group; HT group: high-temperature group; NC group: negative control group; TM group: treatment group. *: *p* < 0.05; **: *p* < 0.01; ns: no significant difference. Scale bars: 1 cm.

**Figure 3:**
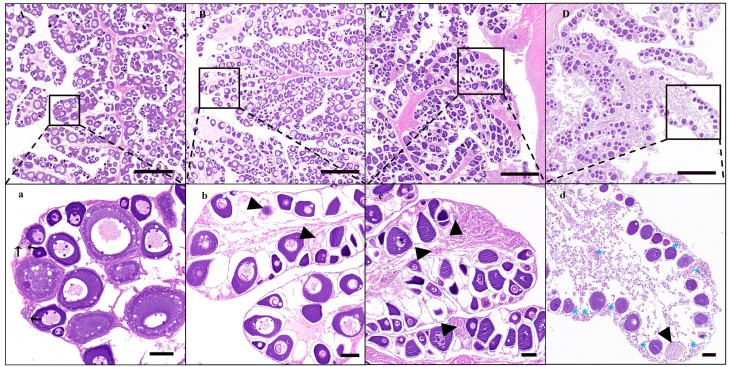
Histological observations of the ovaries of different groups of three-year-old fish. (**A**–**D**) Histology of the NT, HT, NC, and TM groups of three-year-old fish; (**a**–**d**) represent enlarged images corresponding to (**A**–**D**). NT group: natural-temperature group; HT group: high-temperature group; NC group: negative control group; TM group: treatment group. Arrow: oogonia; arrowhead: oocytes in process of ablation; blue asterisk: blood cells. Scale bars of (**A**–**D**): 500 μm; scale bars of (**a**–**d**): 50 μm.

Corresponding to the different treatment methods, the ovarian tissues showed varying degrees of atrophy, ranging from congestion (Figure 2B), a little shriveled (Figure 2D), to completely shriveled (Figure 2F) in comparison with the NT groups of one-, two-, and three-year-old fish (Figure 2A,C,E). These effects were most obvious in the three-year-old TM group. Furthermore, the GSI of this group was significantly lower than that of the NT, HT, and NC groups of the same-aged fish (*p* < 0.01, *p* < 0.05, *p* < 0.01). The GSI values in the one- and two-year-old TM groups were also decreased but did not differ significantly from the values of the other groups of the same ages (*p* > 0.05, Figure 2G). Further histological results showed that the gonad development stages of one-, two-, three-year-old fish in NT groups were at II (Figure S1A,a), the end of II (Figure S2A,a), and III (Figure 3A,a), respectively. After treatment with busulfan and a high temperature of 28 °C, oogonia cells had disappeared, the oocytes showed varying degrees of apoptosis, and the spawning plate had shrunk in all HT and NC groups; however, these effects were less marked in the HT and NC groups in comparison to TM groups of the same ages (Figure 3B,C,b,c, Figures S1B,C,b,c and S2B,C,b,c). Fish in the three-year-old TM group showed the greatest amount of spawning plate atrophy, with enlargement of the entire ovarian lacuna, few oocytes on the spawning plate, and higher levels of oocyte apoptosis compared with the baseline; furthermore, oocytes undergoing apoptosis and blood stasis could also be seen on the spawning plate (Figure 3D,d). Although the degree of apoptosis in the one- and two-year-old TM groups (Figures S1D,d and S2D,d) was more obvious than that of the other groups of the same age, the levels of apoptosis were markedly lower than that seen in the three-year-old TM group (Figure 3D,d).

**Figure 4:**
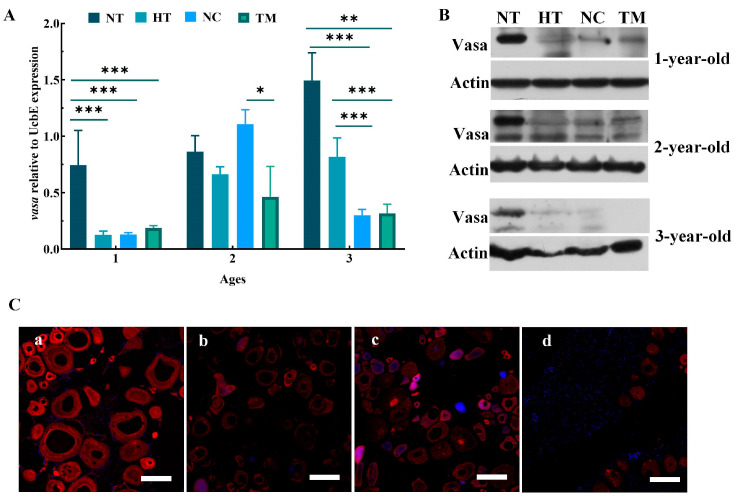
*Vasa* expression in different groups. (**A**) *Vasa* mRNA expression relative to *UcbE*, measured by qRT-PCR. (**B**) Vasa protein expression relative to Actin, shown by Western blotting. (**C**) Anti-Vasa immunofluorescence in the NT (**a**), HT (**b**), NC (**c**), and TM (**d**) groups in three-year-old fish. NT group: natural-temperature group; HT group: high-temperature group; NC group: negative control group; TM group: treatment group. ns: no significant difference; *: *p* < 0.05; **: *p* < 0.01; ***: *p* < 0.001. Scale bars: 50 μm.

These results were verified by examining the mRNA and protein expression of *vasa*. The expression levels of *vasa* mRNA in the TM groups were significantly lower than those of the NT groups, in both one- and three-year-old fish (*p* < 0.001). In two-year-old fish, although the mRNA levels were reduced, the difference was only significant compared with the NC group (*p* < 0.05) and not significant compared with NT and HT groups (Figure 4A). Western blotting also showed that compared with the NT group, Vasa protein expression levels in the HT, NC, and TM groups were reduced compared with the NT group at the same age, especially in the TM group of three-year-old fish (Figure 4B). Lastly, Vasa immunofluorescence was analyzed in the gonadal tissues of the different groups of three-year-old fish, and it was found that there was only sporadic weak red fluorescence in the TM group compared with NT, HT, and NC groups, indicating significant apoptosis of endogenous germ cells (Figure 4C).

### 3.3. SSC Identification

**Figure 5:**
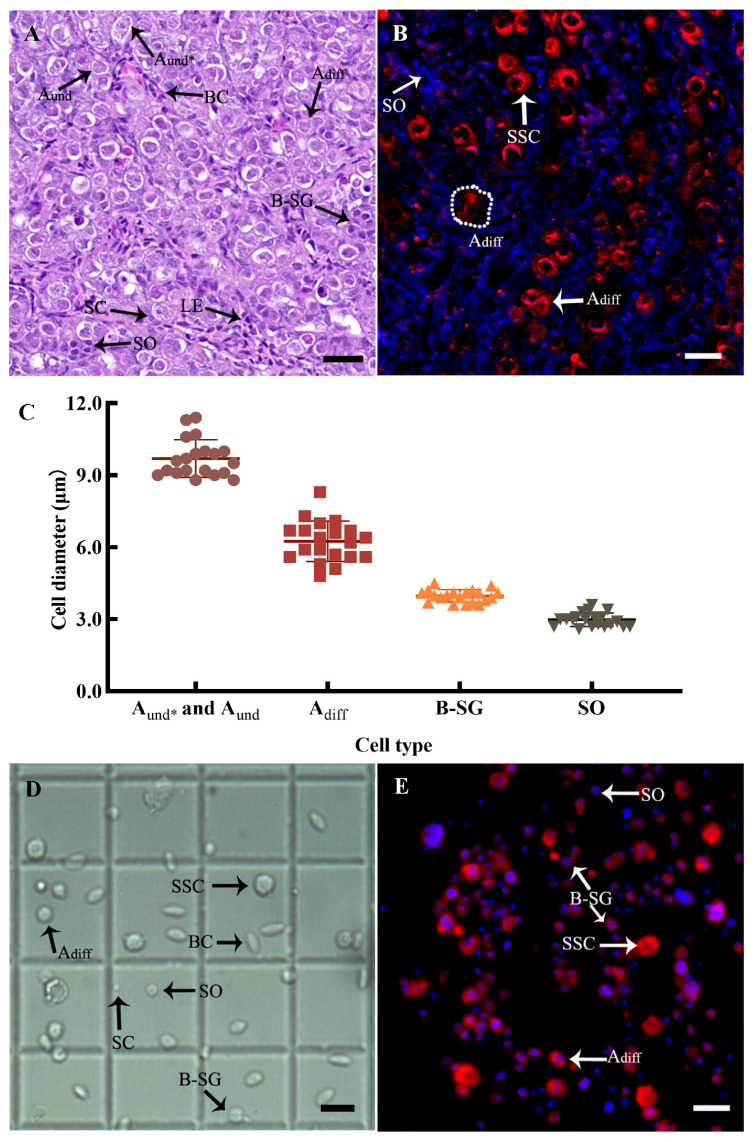
Identification and isolation of SSCs. (**A**) Histology of the testes of one-year-old donor fish. (**B**) Anti-Vasa immunofluorescence of testis tissue from one-year-old donor fish. (**C**) Diameters of spermatogenic cells of different grades. (**D**) Microscopic observations of SSC isolation. (**E**) Anti-Vasa immunofluorescence in isolated SSCs. A_und*_ and A_und_: undifferentiated spermatogonia, collectively known as SSC_S_; A_diff_: differentiated spermatogonia; B-SG: B-type spermatogonia; SO: primary spermatocyte; SC: Sertoli cell; BC; blood cell; LE, Leydig cell. Scale bars: 20 μm.

It was found that the testes of the donors at the end of stage II had large numbers of spermatogonia, predominantly A_und*_, A_und_, and A_diff_ spermatogonia cells, followed by type B spermatogonia and a small number of primary spermatocytes, as shown by histological observation (Figure 5A) and anti-Vasa immunofluorescence (Figure 5B). A_und*_ and A_und_ spermatogonial cells, collectively classified as SSCs, were the largest germ cells in the testis, had 1–2 dense nucleoli with a higher cytoplasm ratio than other germ cells, and existed in the form of a single cell in each seminal vesicle, which had the capacity for self-renewal and differentiation. A_diff_ spermatogonial cells were smaller than SSCs and appeared in pairs with 2–8 cells in each seminal vesicle. There were 16–200 type B spermatogonia cells in each cyst and they showed more heterochromatin. Vasa protein was strongly expressed in the above spermatogonia and barely expressed in spermatocytes. At the same time, the diameters of different spermatogenic cells were also distinguishable, and the statistical results showed that the diameters of SSCs, A_diff_ spermatogonia, type B spermatogonia, and primary spermatocytes were 9.70 ± 0.18 μm, 6.25 ± 0.19 μm, 3.98 ± 0.06 μm, and 2.98 ± 0.06 μm, respectively (Figure 5C), which were subsequently used as the basis for the isolation and purification of SSCs combined with Vasa protein expression analysis.

### 3.4. SSC Isolation and Purification from Donor Testes

After enzymatic digestion, filtration, and cleaning, the cell survival rate was 91.67 ± 0.88%, with an SSC proportion of 18.21 ± 2.77%, which was converted to an average of (3.38 ± 0.51) × 10^4^ SSCs per milligram of testis tissue (Table 3). The SSC identification was further confirmed by cell diameter and anti-Vasa immunofluorescence (Figure 5D,E).

**Figure 6:**
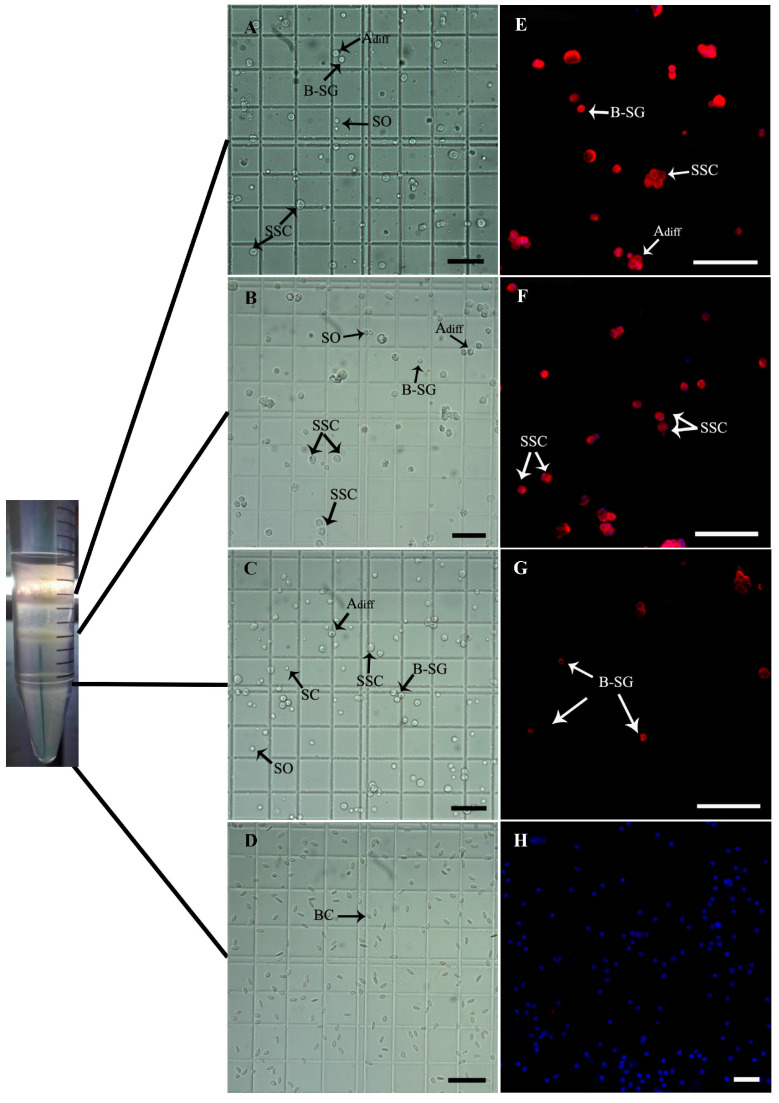
Purification of SSCs. (**A**–**D**) Microscopic observations of cells from layers 1–4 from Percoll density gradient centrifugation. (**E**–**H**) Anti-Vasa immunofluorescence of cells from layers 1–4 from the Percoll density gradient centrifugation. A_diff_: differentiated spermatogonia; B-SG: type B spermatogonia; SO: primary spermatocyte; SC: Sertoli cell; BC; blood cell. Scale bars: 20 μm.

Four bands of cells were obtained after Percoll density gradient centrifugation. These, apart from the fourth band, were then analyzed (Figure 6, Table 3). The survival rates of cells from layers 1 to 3 were 90.57 ± 2.03%, 86.68 ± 1.01%, and 88.82 ± 2.20%, respectively, indicating no significant reduction in survival compared with that before purification (*p* > 0.05, *p* > 0.05, *p* > 0.05). The cells in layers 1 and 2 were mainly SSCs, A_diff_ spermatogonia, and type B spermatogonia (Figure 6A,B,E,F), and the layer 3 cell band contained mainly spermatogenic cells which tended to differentiate and Sertoli cells (Figure 6C,G), while the cells in the fourth layer were mostly blood cells (Figure 6D,H). The SSC proportions of layers 1 and 2 were 64.35 ± 3.50% and 61.83% ± 7.16%, respectively, both of which were significantly increased relative to the pre-purification values (*p* < 0.001, *p* < 0.001). However, the proportion of SSCs in the third layer did not change significantly from that before purification (*p* > 0.05, Table 3). Thus, the upper two cell layers were able to be used for transplantation as they showed no significant loss compared to the pre-purification numbers of SSCs obtained per milligram of tissue (*p* > 0.05, Table 3).

### 3.5. SSC Transplantation and Fate in the Ovaries of Sterile Recipients

**Figure 7:**
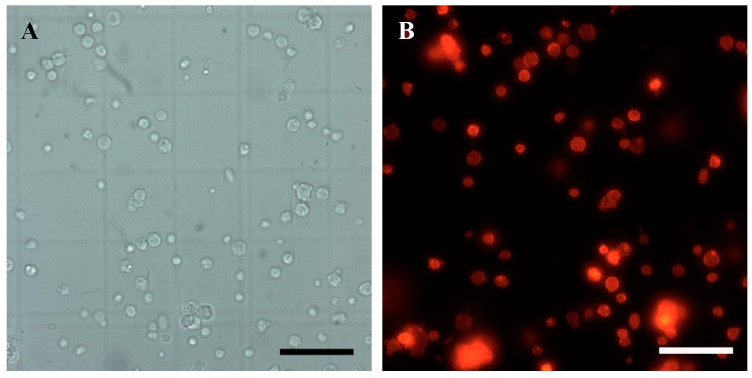
The donor SSCs labeled PKH26. White light (**A**) and fluorescence (**B**) observations of PKH26-labeled spermatogonia. Scale bars: 50 μm.

**Figure 8:**
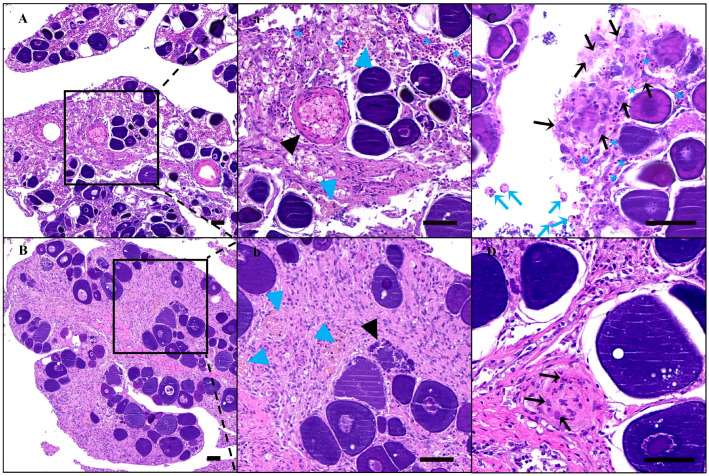
The recovery of ovaries after transplantation. Histology of recipient ovaries on the 11th day (**A**,**a**,**C**) and 21st day (**B**,**b**,**D**) after transplantation. (**a**,**b**) are enlarged images corresponding to the contents of the boxes of (**A**,**B**). (**C**,**D**) highlight the status of germ stem cells of recipients on the 11th day and 21st day after transplantation. Blue asterisk: blood cells; black arrowhead: oocytes in process of ablation; blue arrowhead: hemosiderin pigmentation; black arrow: normal germ stem cells (GSCs); blue arrow: abnormal GSCs. Scale bars: 50 μm.

**Figure 9:**
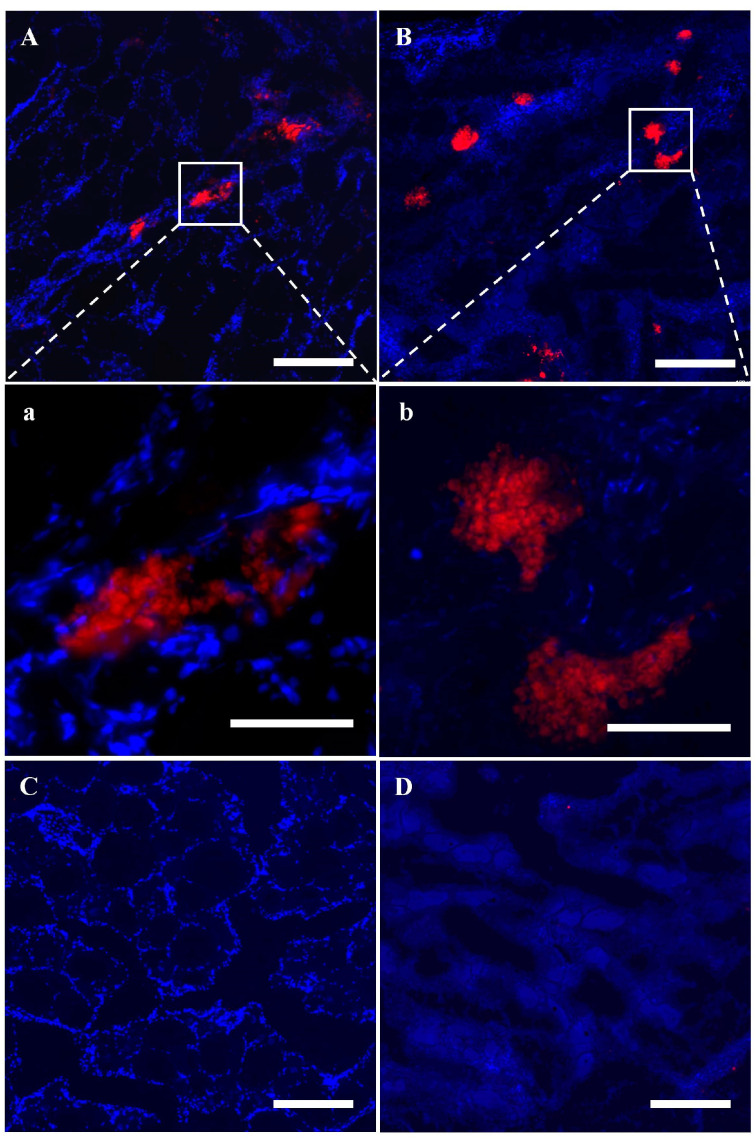
Colonization and proliferation of PKH26-labeled SSCs in recipients. (**A**) Colonization of GSCs in recipient ovaries on 11th day after transplantation. (**B**) Proliferation of GSCs in recipient ovaries on 21st day after transplantation. (**a**,**b**) are enlarged images corresponding to (**A**,**B**). (**C**,**D**) are the control groups without transplanted PKH26-labeled cells corresponding to (**A**,**B**), respectively. Scale bars of (**A**–**D**): 200 μm. Scale bars of (**a**,**b**): 50 μm.

In order to trace the donor SSC fate in the recipient ovaries, almost all the cell membranes of SSCs were labeled with red fluorescence with PKH26 (Figure 7A,B). And the stained SSCs were then transplanted into recipient ovarian tissue through the urogenital aperture and the fallopian tube using an enema tube. On the 11th day after transplantation, there were still significant extravasated blood, hemosiderosis symptoms, and ablated oocytes in the ovaries and the spawning plates were also loose (Figure 8A,a). Fortunately, donor cells labeled with PKH26 successfully colonized in the recipient germ cell nests mainly composed of granulosa cells in which the endogenous germ cells had been lost or dying and were near the stromal cells and blood vessels (Figure 9A,a), while there was no red fluorescence in the non-transplanted ovarian tissue (Figure 9C). On the 21st day after transplantation, the texture of spawning plates became denser and the symptoms of extravasated blood had basically disappeared, with only a small amount of hemosiderosis (Figure 8B,b). At the same time, donor germ cells had formed new stem cell niches and proliferated into cell clusters, which were similar to oogonial nests derived from oogonium multiplied by mitotic divisions, and red fluorescence was not diminished at all (Figure 9B,b). Similarly, no red fluorescence was observed in the non-transplanted gonads at the same period (Figure 9D). Histological observation further corroborated the above result, some donor SSCs colonized (Figure 8C) and gradually formed oogonia cell nests in spawning plates (Figure 8D). It was also found that some donor cells failed to colonize and were scattered in the ovarian lumen, showing nuclear contraction or cell membrane rupture on 11th day after transplantation (Figure 8C).

## 4. Discussion

Donor progeny can be obtained in the next breeding season by the transplantation of germ cells into adult fish [16,33,34]. The necessary condition for the adult fish as recipients was sterility, and the most common method used for the induction of sterility is growth under high-temperature conditions combined with multiple injections of busulfan, which were documented to have pathological results in fish [11,15,23,27,35]. The ovaries of the young *P. olivaceus* were previously observed to be discolored, with adhesion to the visceral mass after germ cell depletion by intraperitoneal injection of either 80 mg/kg or 120 mg/kg of busulfan, suggesting that these fish would not be successful recipients [20]. During the same period, we injected two doses of 18 mg/kg of busulfan into the testes through the vas deferens without causing any adverse effects such as skin ulceration in *P. olivaceus* [16]. These results encouraged us to optimize the technical route for female recipient preparation in *P. olivaceus*. Thus, in the present study, three methods of inducing apoptosis in female endogenous germ cells were compared for the preparation of suitable *P. olivaceus* recipients. The best effects on germ cell apoptosis were observed in three-year-old fish after four intraovarian injections of 20 mg/kg of busulfan after the spawning season, with minimal changes in body weight before and after the experiment and no difference in survival rate from the other two depletion methods. Although the oogonia and most of the oocytes were apoptotic, a small number of ablating and normal oocytes were still present in the ovarian tissue of three-year-old fish, which was due to their large size or the presence of yolk.

In addition to the preparation of endogenous germ-cell-depleted recipients, another key factor for successful SSCT is the obtaining of high SSC yields. Different levels of spermatogenic cells can easily be distinguished by the size of the cell or nucleus and the number of germ cells in the seminiferous vesicle. Each SSC, containing A_und*_ and A_und_ spermatogonia, is wrapped in a single seminal vesicle, significantly larger than the surrounding cells, and has 1–2 dense nucleoli and a high nucleoplasmic ratio [36,37]. A_diff_ spermatogonia, which are usually found in pairs of two, four, or eight within the seminiferous vesicle and are much smaller than SSCs, undergo several divisions before their formation into type B spermatogonia and the initiation of meiosis [38,39]. At the same time, Vasa is a molecular marker specific to germ cells, which is mainly expressed in spermatogonia in testes. Therefore, we calculated the diameters of spermatogenic cells of different levels, assessed according to their specific characteristics, thus providing a reference for the isolation and purification of SSCs. Because the proportion of SSCs varies with the gonad development cycle [36], it was considered preferable to select the testes at or before stage II. The testes of 12-month-old fish remain at stage II, with a higher proportion of SSCs, allowing the isolation of sufficient numbers of SSCs for transplantation compared to fish of earlier ages. The enzymes used for the isolation of spermatogonia are collagenase, trypsin, or combinations of multiple digestive enzymes [10,33,40]. The methods used for the purification of SSCs include immunomagnetic beads [41], flow separation [42], and Percoll density gradient centrifugation [43]. The testes of *P. olivaceus* are relatively soft and thus easy to digest. Meanwhile, the use of Percoll gradients was more convenient as it did not require the preparation of specific antibodies, flow cytometry, and other specific processes, and was also less damaging to the cells. Therefore, in the present study, we used a milder trypsin digestion of the tissues, ultimately obtaining sufficient SSCs, with a high percentage of more than 60.00%, using Percoll gradients.

Single-sex farming is of great importance in fish culture. There is a large gender difference in the growth rate and commercial value of fish. In addition, single-sex culture can improve breeding efficiency and reduce breeding costs, which has prompted breeders to consider sex-controlled breeding as an important research area [44]. In addition to the techniques of gynogenesis, androgenesis, and sexual reversal, GCT has also been used in fish sex-controlled breeding [12]. It takes three generations to achieve all-male breeding through pseudo-female induction [45] and the very low induction rate of androgenesis hinders the breeding of all-male germplasm [31,46]. At the same time, although the use of gynogenesis can rapidly yield all-female seedlings, the intergenerational transmission of desirable male traits is often ignored. The use of GCT technology can effectively solve these problems, essentially because GSCs are pluripotent and can differentiate into gametes of both genders, as has been shown in many fish [9,12,15]. In this paper, we also preliminarily proved that SSCs could clone and proliferate in the recipient ovaries of *P. olivaceus*. In the next step, we will optimize the procedure of SSCT in female *P. olivaceus* in order to obtain a high proportion of offspring from male donors.

## 5. Conclusions

In conclusion, we comprehensively evaluated and determined the optimal methods for the preparation of sterile female recipients and the isolation and purification of high-yield donor SSCs in *P. olivaceus*, and the SSCs could be cloned and proliferated in allogenic female recipients after transplantation. This study provided technical support for the effective preservation of male traits of *P. olivaceus* by SSCT.

## Figures and Tables

**Table 1:** The body weight of fish before experiment.

Gender	Recipients (♀)			Donors (♂)
Age	1	2	3	1
Body weight (g)	340.00 ± 26.94	960.00 ± 52.55	1480.00 ± 41.75	262.36 ± 24.05
Gonadosomatic index (GSI)	0.59 ± 0.02	1.03 ± 0.10	1.21 ± 0.10	0.08 ± 0.00
Gonadal development period	Phase II	End of phase II	Phase III	Phase II

**Table 2:** The primer sequences of real-time PCR.

Primer Name	Primer Sequences (5′–3′)	Amplicon (bp)
*vasa*-F	TAGTTCCCTCGTGGTTAGAAGAGT	133
*vasa*-R	GCTGTGCTGTCCTGAGAGAATC	
*UbcE*-F	TTACTGTCCATTTCCCCACTGAC	127
*UbcE*-R	GACCACTGTGACCTCAAGATG	

**Table 3:** Comparison of cell survival rates/SSC proportion/SSC counts before and after enrichment.

Cell Suspension	Cell Survival Rate (%)	SSC Proportion (%)	SSC Counts (×10^4^/mg Testis Tissue)
Before enrichment	91.67 ± 0.88 ^a^	18.21 ± 2.77 ^a^	3.38 ± 0.51 ^a^
1	90.57 ± 2.03 ^a^	64.35 ± 3.50 ^b^	1.52 ± 1.11 ^a^
2	86.68 ± 1.01 ^a^	61.83 ± 7.16 ^b^	
3	88.82 ± 2.20 ^a^	14.60 ± 1.34 ^a^	

Note: different letters indicate significant differences among the groups (*p* < 0.001), while the same letter indicates the absence of a significant difference (*p* > 0.05).

## Data Availability

Data presented in this study are available upon request from the corresponding author.

## Supplementary Materials

The following supporting information can be downloaded at: https://www.mdpi.com/article/10.3390/ani14192887/s1, Figure S1: Histological observations of the ovaries of different groups of one-year-old fish; Figure S2: Histological observations of the ovaries of different groups of two-year-old fish.
