# Supplementary material for: Evaluation of Female Recipient Infertility and Donor Spermatogonial Purification for Germ Cell Transplantation in Paralichthys olivaceus
Source: Animals (Basel). 2024 Oct 8;14(19):2887. doi: 10.3390/ani14192887 (PMC11476266; doi:10.3390/ani14192887)
Supplement: Supplementary file 1 [file animals-14-02887-s001.zip › animals-3188071-supplementary.pdf]

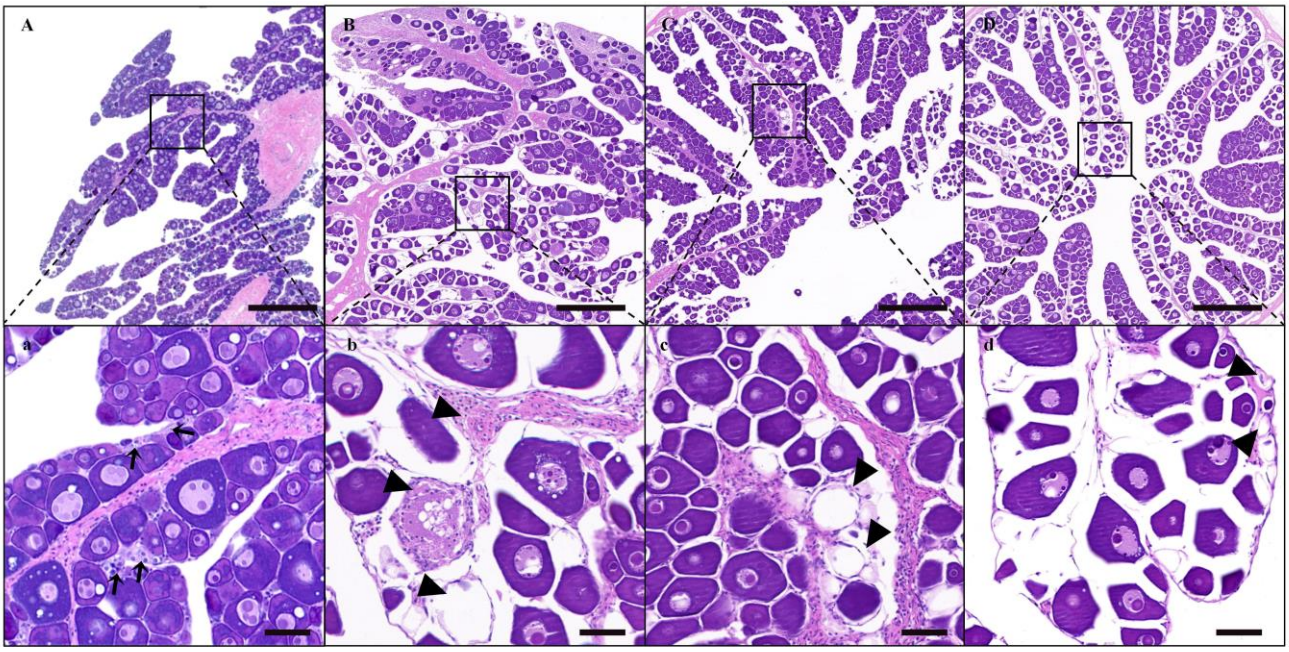

Figure S1. Histological observations of the ovaries of different groups of one-year-old fish. (A-D) Histology of the NT, HT, NC and TM groups of one-year-old fish; (a-d) represent enlarged images corresponding to (A-D). NT group: natural-temperature group; HT group: high-temperature group; NC group: negative control group; TM group: treatment group. Arrow: oogonia; arrowhead: oocytes in process of ablation. Scale bars of A-D: 500 μm; scale bars of a-d: 50 μm.

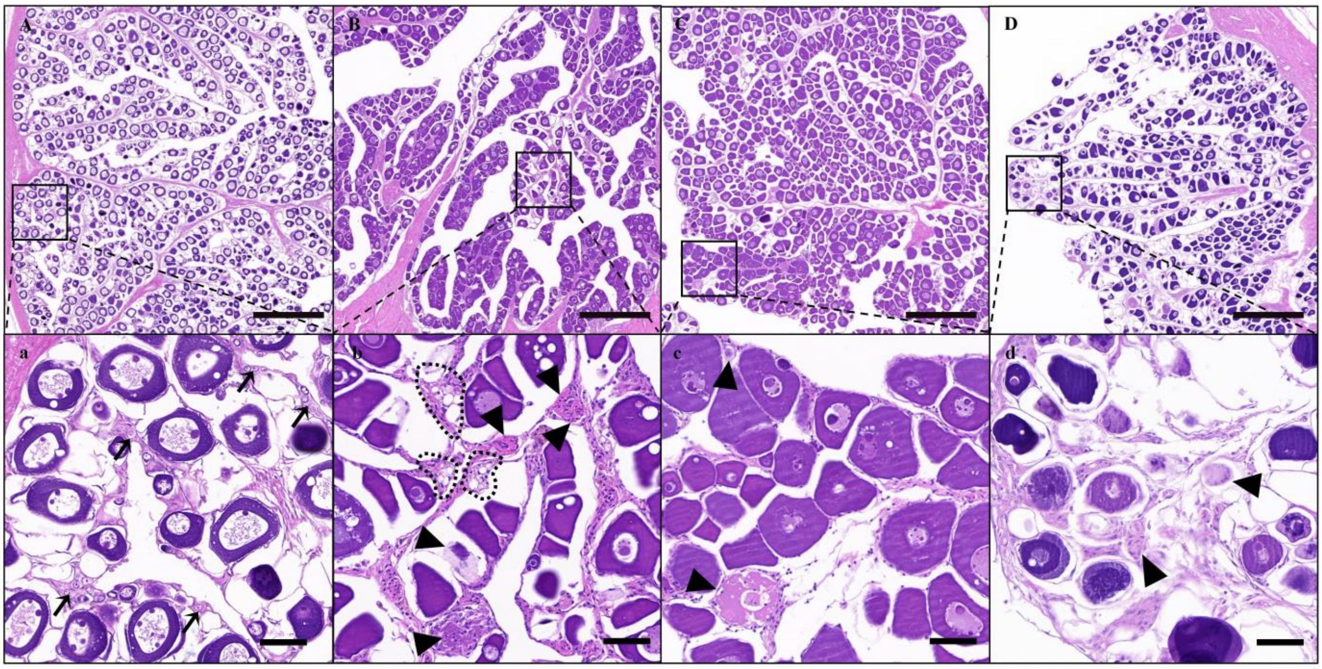

Figure S2. Histological observations of the ovaries of different groups of two-year-old fish. (A-D) Histology of the NT, HT, NC and TM groups of two-year-old fish; (a-d) represent enlarged images corresponding to (A-D). NT group: natural-temperature group; HT group: high-temperature group; NC group: negative control group; TM group: treatment group. Arrow: oogonia; arrowhead: oocytes in process of ablation. The area circled by the dotted line

in the ovaries indicates where oogonia have died. Scale bars of A-D: 500 μm; scale bars of a-d: 50 μm.
