# Supplementary figures and images for: Evaluation of Female Recipient Infertility and Donor Spermatogonial Purification for Germ Cell Transplantation in Paralichthys olivaceus
Source: Animals (Basel). 2024 Oct 8;14(19):2887. doi: 10.3390/ani14192887 (PMC11476266; doi:10.3390/ani14192887)

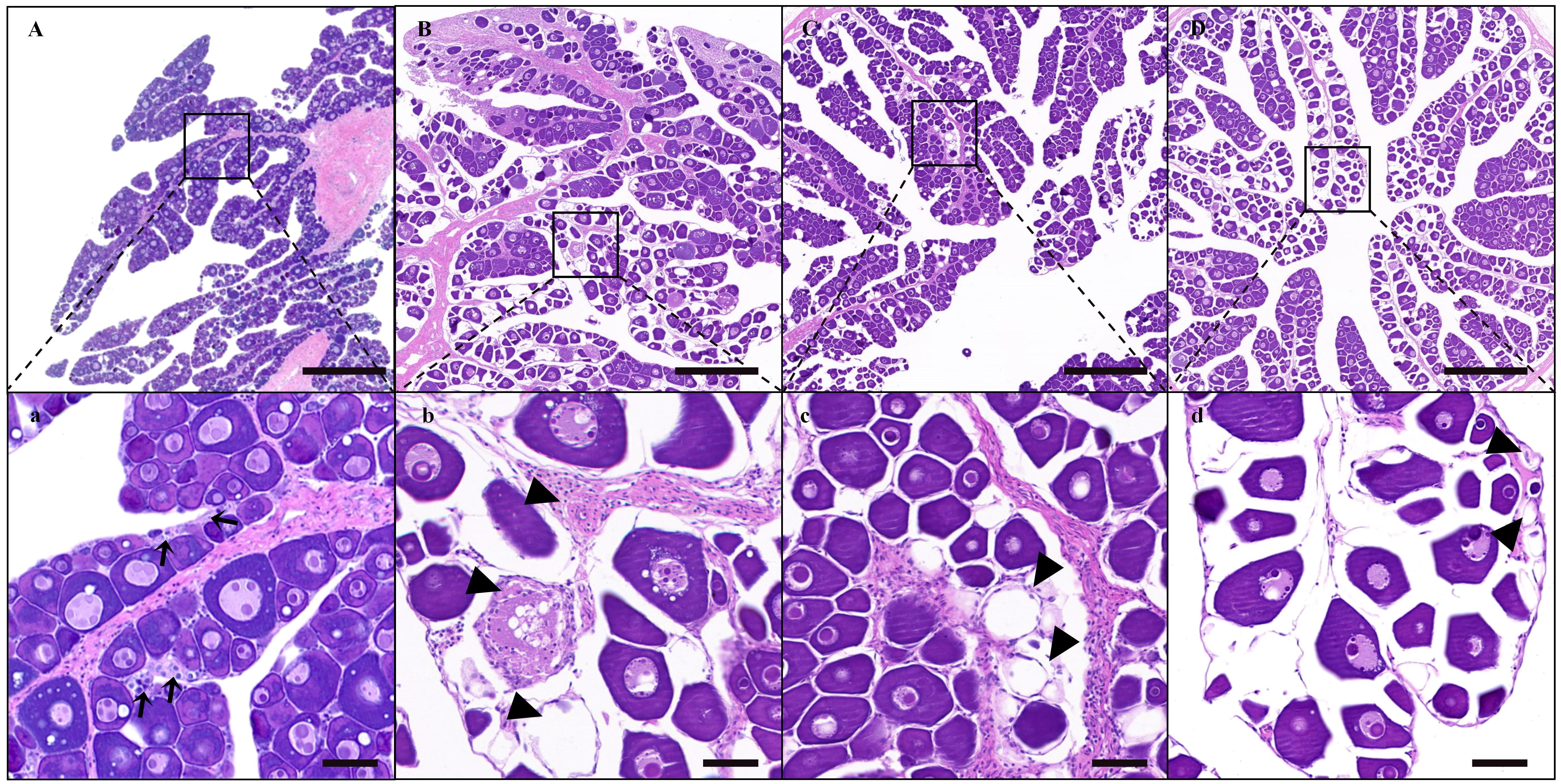

Supplement: Supplementary file 1 [file animals-14-02887-s001.zip › Figure S1.jpg]

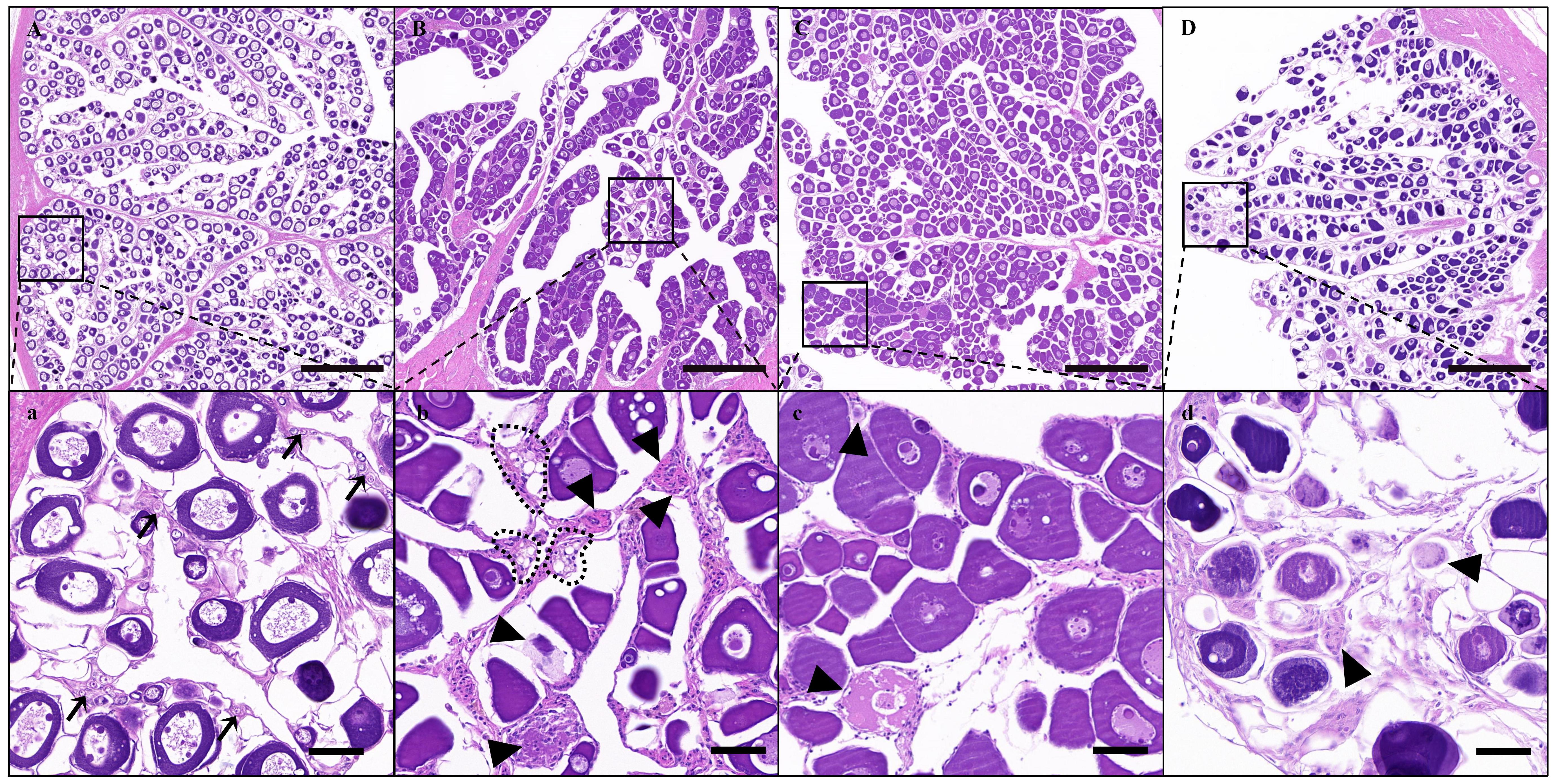

Supplement: Supplementary file 1 [file animals-14-02887-s001.zip › Figure S2.jpg]
